# Non-homologous end joining in class switch recombination: the beginning of the end

**DOI:** 10.1098/rstb.2008.0196

**Published:** 2008-11-13

**Authors:** Ashwin Kotnis, Likun Du, Chonghai Liu, Sergey W. Popov, Qiang Pan-Hammarström

**Affiliations:** Division of Clinical Immunology, Department of Laboratory Medicine, Karolinska Institutet at Karolinska University Hospital Huddinge14186 Stockholm, Sweden

**Keywords:** class switch recombination, non-homologous end joining, alternative NHEJ, microhomology

## Abstract

Immunoglobulin class switch recombination (CSR) is initiated by a B-cell-specific factor, activation-induced deaminase, probably through deamination of deoxycytidine residues within the switch (S) regions. The initial lesions in the S regions are subsequently processed, resulting in the production of DNA double-strand breaks (DSBs). These breaks will then be recognized, edited and repaired, finally leading to the recombination of the two S regions. Two major repair pathways have been implicated in CSR, the predominant non-homologous end joining (NHEJ) and the alternative end-joining (A-EJ) pathways. The former requires not only components of the ‘classical’ NHEJ machinery, i.e. Ku70/Ku80, DNA-dependent protein kinase catalytic subunit, DNA ligase IV and XRCC4, but also a number of DNA-damage sensors or adaptors, such as ataxia–telangiectasia mutated, γH2AX, 53BP1, MDC1, the Mre11–Rad50–NBS1 complex and the ataxia telangiectasia and Rad3-related protein (ATR). The latter pathway is not well characterized yet and probably requires microhomologies. In this review, we will focus on the current knowledge of the predominant NHEJ pathway in CSR and will also give a perspective on the A-EJ pathway.

## 1. Introduction

Immunoglobulin M (IgM) is the primordial antibody class and has been supplemented during evolution by antibody classes (IgG, IgA and IgE) with improved, more specialized, effector functions. The change in antibody class is effectuated by a looping out–deletion–recombination process called class switch recombination (CSR), where the constant region gene of the μ heavy chain (*Cμ*) is replaced by a downstream constant region gene (*Cγ*, *Cα* or *Cϵ*; Stavnezer *et al*. 2008). The process requires germ line (GL) transcription of the unrearranged C region genes (Stavnezer 1996; Chaudhuri & Alt 2004) and is initiated by a B-cell-specific factor, activation-induced cytidine deaminase (AID; Muramatsu *et al*. 2000; Revy *et al*. 2000). The modes of action of AID are discussed elsewhere in this issue.

CSR involves DNA regions, called ‘switch (S) regions’, that are located in the introns upstream of each C region gene, except *Cδ*. S regions are composed of tandemly repeated sequences that contain common pentameric sequences (GAGCT and GGGCT), but differ in the overall length of the repetitive region, the actual sequence of the repeats and the number of polymorphic alleles (Pan-Hammarstrom *et al*. 2007). In both humans and mice, Sμ, Sα and Sϵ are closely related and characterized by a dense clustering of pentameric repeats, with or without a higher ordered structure. The Sγ regions, however, share very little homology with the respective Sμ regions (Pan-Hammarstrom *et al*. 2007).

The initial lesions introduced by AID in the S regions are subsequently processed, leading to the production of DNA double-strand breaks (DSBs; discussed elsewhere in this issue). There are two major mechanisms for the repair of DSBs, homologous recombination (HR) and non-homologous end joining (NHEJ). The former is dependent on sequence homology and is the most active in the late S/G2 phase. The latter uses little or no sequence homology, which is sometimes imprecise and functions throughout the cell cycle. NHEJ is therefore considered to be the principal mechanism used in CSR, as AID-dependent DSBs are introduced and repaired mainly in the G1 phase of the cell cycle (Schrader *et al*. 2007) and the nature of S region sequences (lack of long stretches of perfect homology between the different S regions) would theoretically not support HR.

The ‘classical’ NHEJ machinery requires a number of factors, including Ku70/Ku80, DNA-dependent protein kinase catalytic subunit (DNA-PKcs), DNA ligase IV, XRCC4, Artemis and XLF (Cernunnos; Lieber 2008). During the past decade, evidence has accumulated that many of these factors are actually required for the CSR process. When the classical NHEJ pathway is impaired, an alternative, or back-up, NHEJ pathway is operative. In this paper, we will review the current knowledge of the predominant NHEJ pathway in CSR and give a perspective on the alternative end-joining (A-EJ) mechanism.

## 2. The predominant non-homologous end-joining pathway during class switch recombination

The predominant end-joining pathway used in normal cells during CSR requires not only components of the classical NHEJ machinery, but also a number of factors that are considered being DNA-damage sensors, transducers or adaptors. Table 1 summarizes the CSR phenotype in mouse knockout and human disease models. A reduced efficiency of CSR and/or an increased usage of microhomology at recombinational junctions are common features when the predominant NHEJ pathway is impaired (table 1).

### (a) Ataxia-telangiectasia mutated signalling

#### (i) ATM

ATM is a phosphoinositol 3-kinase-like kinase (PIKK) that plays a central role in orchestrating a network of cellular responses to DSBs, including cell cycle control, DNA repair and apoptosis (Shiloh 2003; Lavin & Kozlov 2007). ATM deficiency in humans results in a rare, multi-system disorder, ataxia–telangiectasia (A–T), characterized by cerebellar degeneration with ataxia, telangiectasia, chromosomal instability, radiosensitivity and cancer predisposition (Chun & Gatti 2004).

**Figure 1:**
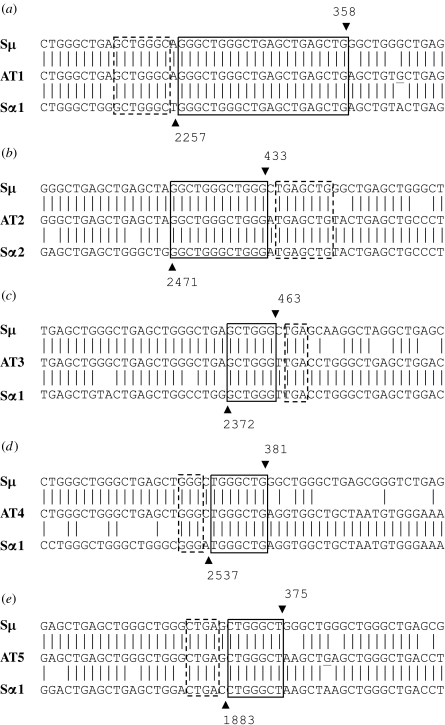
The sequences of the first five Sμ–Sα junctions derived from A–T patients (Pan *et al*. 2002). The Sμ and Sα1 or Sα2 sequences are aligned above and below the recombined switch junctional sequences. Microhomology was determined by identifying the longest region at the S junction of perfect uninterrupted donor/acceptor identity (solid-line boxes). Imperfect repeats were determined by identifying the longest overlap region at the S junction by allowing one mismatch on either side of the breakpoint (the extra nucleotide beyond the perfect matched sequence identity is indicated by dotted-line boxes). The Sμ and Sα breakpoints for each S fragment are indicated by a downward arrowhead and an upward arrowhead, respectively, and their positions in the germ-line sequences (X54713, L19121 and AF030305; Mills *et al*. 1990; Islam *et al*. 1994; Pan *et al*. 2001) are indicated above or below the arrowhead. (*a*) 21 bp microhomology (28/29 bp imperfect repeat), (*b*) 12 bp microhomology (19/20 bp imperfect repeat), (*c*) 6 bp microhomology (9/10 bp imperfect repeat), (*d*) 7 bp microhomology (10/11 bp imperfect repeat), (*e*) 7 bp microhomology (11/12 bp imperfect repeat).

A–T is also recognized as a primary immunodeficiency disorder, with both the cellular and humoral immune systems being affected, leading to recurrent and severe infections (Lavin & Shiloh 1999). IgA deficiency (IgAD) has been observed in 60–80% of patients, and a subgroup suffers from concomitant IgG subclass deficiency (Lavin & Shiloh 1999). During investigations aimed at elucidating the underlying cause of antibody deficiency in A–T patients, we observed that the Sμ–Sα recombination junctions in patient B cells were characterized by a strong dependence on short, perfectly matched sequence homologies (microhomologies) and devoid of normally occurring mutations around the breakpoints (Pan *et al*. 2002). More than 60 per cent of the junctions exhibited a microhomology of 4 bp or more with the longest being 21 bp, as illustrated in figure 1 in the very first set of breakpoints we obtained. This is far beyond what one would expect as a consequence of NHEJ, which should theoretically result in no, or a 1–3 bp, microhomology. The serendipitous finding of an aberrant pattern of Sμ–Sα junctions from A–T patients led us to hypothesize that ATM is involved in the predominantly used NHEJ pathway in CSR. In A–T patients, where ATM function is impaired, the S regions are joined by an A-EJ pathway involving microhomology (Pan *et al*. 2002). This concept was not fully accepted at that time, as ATM was not considered to be a part of the DSB repair machinery, in particular not with NHEJ. Furthermore, definitive evidence showing that NHEJ is involved in CSR was still missing.

ATM-deficient mice have been generated in several laboratories and these mice are characterized by growth retardation, infertility, radiosensitivity and development of thymic lymphomas (Barlow *et al*. 1996; Elson *et al*. 1996; Xu *et al*. 1996). The serum level of immunoglobulins is, however, largely normal (Xu *et al*. 1996) although reduced serum titres of antigen-specific IgG and IgA antibodies have been detected after immunization (Lumsden *et al*. 2004). Two studies have subsequently shown that B cells from ATM-deficient mice cannot switch to IgG and IgA as efficiently as do wild-type B cells, suggesting an intrinsic B-cell defect during CSR (Lumsden *et al*. 2004; Reina-San-Martin *et al*. 2004). Furthermore, the defect is not due to alterations in cell proliferation, GL transcription or 53BP1 focus formation and has thus been suggested to be located either at an early step referred to as synapsis, i.e. holding the DNA ends in proximity to facilitate subsequent repairs (Reina-San-Martin *et al*. 2004), or at the break repair step (Lumsden *et al*. 2004).

One of the two studies (Lumsden *et al*. 2004) has also shown a significant increase in the length of microhomology at the Sμ–Sγ1 junctions (2.6 bp versus 1.2 bp in wild-type cells), not as dramatic as the Sμ–Sα junctions derived from A–T patients (7.2 bp versus 1.8 bp in controls), but very similar to the Sμ–Sγ junctions from these patients (2.5 bp versus 1.2 bp in controls; Pan *et al*. 2002). As we have discussed previously (Pan-Hammarstrom *et al*. 2007), and will present in more detail later, Sμ–Sα and Sμ–Sγ junctions are resolved differently both under normal circumstances and in patients with various defects in their DNA repair systems. Owing to the higher degree of homology between Sμ and Sα when compared with Sμ and Sγ, the microhomology-based pathway would be a more attractive alternative for Sμ/Sα recombination when the classical NHEJ pathway is impaired. Thus, we would probably observe a more dramatic shift in the use of longer microhomologies in ATM-deficient mouse B cells if the Sμ–Sα junctions were analysed. It is currently unclear though, whether a ‘normal’ appearance of Sμ–Sγ junctions, i.e. a normal length of microhomology, would simply indicate a normal end joining or whether it may actually result from yet another A-EJ mechanism that does not require a long microhomology.

**Figure 2:**
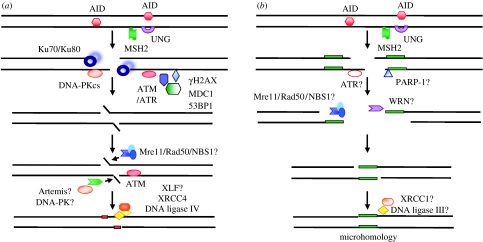
Hypothetical model for the end-joining mechanisms during CSR. AID initiates CSR, probably through deamination of deoxycytidine (dC) residues in the S regions. The dC:dU mismatches can then be processed by either the MSH2-dependent mismatch repair pathway or the UNG-dependent base excision repair, leading to the production of DSBs in the S regions. In the predominant NHEJ pathway, Ku70/Ku80 binds to DNA ends and recruits and activates DNA-PKcs. They are probably important for the synapsis process. ATM and ATM-dependent factors γH2AX, MDC1 and 53BP1 are required for the predominant NHEJ pathway, probably at the synapsis or end-activation step. Together they may be configurating the DNA termini for subsequent repair steps and/or regulating the cell cycle response. ATM may also have a direct role in the end-processing step by phosphorylation of Artemis, a nuclease that may have the potential to repair a subset of DSBs in CSR. The Mre11 complex may be involved in CSR either by activating ATM and/or as a nuclease that is required for the microhomology-mediated end joining. Finally, XRCC4/DNA ligase IV and possibly XLF are involved in the ligation step. The factors involved in the A-EJ are not known but a few candidates are highlighted in the figure (indicated by question marks). (*a*) Predominant NHEJ, (*b*) alternative end joining.

The exact function of ATM in the predominant NHEJ pathway in CSR remains unclear. ATM phosphorylates a number of factors that are known to be involved in CSR, including NBS1, Mre11, γH2AX, 53BP1 and MDC1. Thus, one possible role of ATM is to recruit and/or activate other DNA-damage response factors, such as γH2AX, 53BP1, MDC1 and possibly the Mre11 complex, configuring the DNA termini for subsequent repair steps, and/or slowing down cell cycle progression until the repair is complete (Lieber 1999; Lieber *et al*. 2003; figure 2). As we will discuss below, however, the CSR phenotype resulting from deficiency of any of these ATM substrates is clearly not identical to a deficiency of ATM (table 1). A second possibility is that ATM may have a more direct role in the end-processing step through phosphorylation of a proposed nuclease that participates in NHEJ, i.e. Mre11 or Artemis (figure 2). Artemis would be an interesting candidate, as it is a downstream component in the ATM signalling pathway required for the repair of a subset of radiation-induced DSBs, but dispensable for ATM-dependent cell-cycle checkpoint arrest (Riballo *et al*. 2004). Artemis, however, appears to be dispensable for efficient CSR (Rooney *et al*. 2005), although a recent study has provided evidence that Artemis is required for repair of at least part of the CSR-related chromosomal breaks at the Ig locus (Franco *et al*. 2008). Finally, a recent, large-scale proteomic analysis has identified an impressive list of ATM and ATR substrates (*n*=700), including some additional factors that have previously been implicated in CSR, such as the mismatch repair factors MSH2, MSH6, MLH1, PMS2, EXO1 and RPA1 (Matsuoka *et al*. 2007). An increased length of microhomology at Sμ–Sγ junctions has indeed been observed in MLH1 and PMS2 knockout mice (Ehrenstein *et al*. 2001; Schrader *et al*. 2002), supporting a potential link between ATM/ATR and the mismatch repair factors.

#### (ii) γH2AX (phosphorylated H2AX)

In response to DNA DSBs by ionizing radiation, H2AX is rapidly phosphorylated by ATM or DNA-PKcs (Burma *et al*. 2001; Hickson *et al*. 2004), resulting in the formation of γH2AX foci that spread throughout a megabase-long region flanking the break (Rogakou *et al*. 1999). In cells undergoing CSR, NBS1 and γH2AX foci are formed and co-localized at the CH region during the G1 phase of the cell cycle, suggesting that these factors might also have a role in response to CSR-induced DSBs (Petersen *et al*. 2001).

H2AX-deficient mice are growth retarded, immuno- deficient, radiosensitive and show male-specific infertility (Celeste *et al*. 2002). Serum levels of IgG1, IgG3 and IgA are significantly reduced in these mice and *in vitro* CSR to IgG1 and IgG3 is markedly reduced (approx. 25–30% of wild-type levels; Petersen *et al*. 2001; Celeste *et al*. 2002). The impaired CSR in H2AX-deficient cells is not due to an alteration in induction of GL transcription and short-range intra-switch region recombination proceeds normally (Reina-San-Martin *et al*. 2003). The pattern of Sμ–Sγ1 junctions is also largely normal, with a normal length of microhomology at the junctions and only a slightly reduced frequency of mutations around the junctions (Reina-San-Martin *et al*. 2003). The role of γH2AX in CSR has therefore been proposed to facilitate long-range synapsis of two different S regions (Reina-San-Martin *et al*. 2003). A recent study has further shown that AID-dependent IgH locus chromosome breaks occur at a high frequency in H2AX-deficient B cells undergoing CSR, suggesting that γH2AX may promote end joining during CSR (Franco *et al*. 2006). Interestingly, activated B cells deficient in ATM, 53BP1 or MDC1 show a similar phenotype, with an increased number of IgH locus breaks and translocations (Franco *et al*. 2006), implying that they may have a common function in the predominant NHEJ pathway in CSR. However, the largely normal pattern of Sμ–Sγ junctions in H2AX-deficient cells argues against the idea but, as we have discussed above, analysis of Sμ–Sα junctions in these cells will be required before it is defined as normal. Alternatively, ATM and γH2AX, or 53BP1 (as discussed below), play additional roles in CSR, which are independent of each other.

#### (iii) MDC1

MDC1, yet another ATM substrate (Goldberg *et al*. 2003; Lou *et al*. 2003; Stewart *et al*. 2003), is a mediator or adaptor that mediates γH2AX-dependent chromatin retention of DNA-damage response factors (Stucki & Jackson 2006). MDC1-deficient mice show similar phenotypes as H2AX-deficient mice, including growth retardation, male infertility, immunodeficiency, chromosomal instability and radiosensitivity (Lou *et al*. 2006). Serum levels of immunoglobulins are normal in MDC1-deficient mice, and a reduction in CSR to approximately 50–75% of wild-type levels has been observed in cultured MDC1-deficient B cells (Lou *et al*. 2006). The CSR junctions were, however, not analysed in these cells.

#### (iv) 53BP1

53BP1 was first identified as a p53-binding protein (Iwabuchi *et al*. 1994) and later shown to participate at an early stage in DNA DSB signalling (Schultz *et al*. 2000; DiTullio *et al*. 2002; Fernandez-Capetillo *et al*. 2002; Wang *et al*. 2002). 53BP1 is phosphorylated by ATM but may also be an activator of ATM in response to DNA DSBs (for review see Mochan *et al*. 2004). 53BP1-deficient mice are growth retarded, radiosensitive, immunodeficient and predisposed to cancer (Ward *et al*. 2003), a phenotype that resembles ATM or H2AX knockout mice. Studies on 53BP1-deficient mice have further demonstrated that 53BP1 is dispensable for HR, variable diversity joining (V(D)J) recombination and somatic hypermutation (SHM) but is clearly required for CSR (Manis *et al*. 2004; Ward *et al*. 2004). Serum levels of IgG and IgA are substantially reduced (Manis *et al*. 2004) and the ability of 53BP1-deficient B cells to switch *in vitro*, to all the isotypes, is severely impaired (reduced to 2–10% of wild-type levels; Manis *et al*. 2004; Ward *et al*. 2004). The CSR defect is not due to a proliferative block or an alteration of GL transcription (Manis *et al*. 2004; Ward *et al*. 2004) and the Sμ–Sγ1 junctions reveal no significant differences in the length of microhomology or mutation frequency (Manis *et al*. 2004; Reina-San-Martin *et al*. 2007). However, unusual long insertions, not seen in 240 S junctions from wild-type cells, have been observed in two Sμ–Sγ1 53BP1^−/−^ junctions, and intra-switch recombination is enhanced in 53BP1-deficient cells (Reina-San-Martin *et al*. 2007), suggesting a potential role of 53BP1 in synapsis or protecting the DNA ends of the two S regions.

There is thus far no human disease that has been linked to mutations in the gene encoding 53BP1. A recent study has, however, described a patient who suffered from the RIDDLE syndrome (radiosensitivity, immunodeficiency, dysmorphic features and learning difficulties; Stewart *et al*. 2007). Cells from this patient lack an ability to recruit 53BP1 to the sites of DNA DSBs. Mutations were neither found in the gene encoding 53BP1 itself nor in the genes encoding ATM, MDC1, H2AX and PR-Set7, factors that are known to be involved in regulating the localization of 53BP1 to the breaks (Stewart *et al*. 2007). It is possible that an as yet unknown protein, acting upstream of 53BP1 during the cellular response to DNA DSBs, is defective in this patient. It is interesting to note that significantly increased microhomology is observed at the Sμ–Sα junctions amplified from this patient, with 94 per cent of junctions exhibiting a microhomology of 4 bp or more (Stewart *et al*. 2007). A reduced frequency of insertions and mutations around the Sμ–Sα breakpoints is also observed. The Sμ–Sγ3 junctions from this patient have also been analysed, albeit unfortunately not shown in the paper. Nevertheless, the altered pattern of Sμ–Sα junctions in this patient suggests that there could be a shift from the dominant NHEJ to the A-EJ pathway when the formation of 53BP1 foci is defective.

The precise role of 53BP1 in CSR remains elusive. The CSR defect observed in 53BP1-deficient mice is more severe than that in ATM- or H2AX-deficient mice, and the pattern in the Sμ–Sγ junctions is different from that of ATM-deficient cells (although Sμ–Sα junctions have not been analysed in 53BP1-deficient mice). It is thus likely that in addition to being part of the ATM signalling pathway, 53BP1 also has a role in CSR, which is independent of ATM or γH2AX. A recent study has shown that MDC1 functions primarily in HR/sister chromatic recombination, in a manner that is strictly dependent upon its ability to interact with γH2AX, whereas 53BP1 functions in an XRCC4-dependent NHEJ pathway, through interaction with the dimethylated lysine 20 of histone H4, a process that is independent of γH2AX (Xie *et al*. 2007). If this is also true for CSR, it would probably explain why CSR is affected in the order of 53BP1^−/−^ more than H2AX^−/−^ more than MDC1^−/−^.

#### (v) The Mre11–Rad50–NBS1 complex

The Mre11 complex is a multisubunit nuclease composed of Mre11, Rad50 and NBS1. This complex is required for telomere maintenance, cell-cycle checkpoint signalling, DNA replication, meiotic recombination and efficient repair of DNA DSBs by HR and/or NHEJ (D'Amours & Jackson 2002; Lavin 2007). The Mre11 complex acts as a sensor of DNA DSBs, localizes to the sites of break and recruits ATM (Berkovich *et al*. 2007; Lavin 2007). This will lead to autophosphorylation and activation of ATM, which in turn will phosphorylate many substrates, including members of the Mre11 complex. The Mre11 complex thus acts both upstream and downstream of ATM. In humans, no mutations in Rad50 have been reported to date. Mutations in genes encoding Mre11 and NBS1 result in two chromosomal instability syndromes, ataxia–telangiectasia-like disorder (ATLD; Stewart *et al*. 1999) and Nijmegen breakage syndrome (NBS; Varon *et al*. 1998), where both exhibit features that are characteristic for A–T.

NBS patients are characterized by immunodeficiency, microcephaly, chromosomal instability and a high incidence of lymphoid malignancies (Digweed & Sperling 2004). Deficiency of IgG and/or IgA is observed in 80–90% of the patients (Gregorek *et al*. 2002), which could be due to a lack of T-cell help and/or an intrinsic B-cell defect. By analysing the *in vivo*-switched B cells, we have previously shown a reduced level of CSR to IgA in NBS patients (Pan *et al*. 2002). The recovered Sμ–Sα and Sμ–Sγ junctions also show a significantly increased length of microhomology, although not as dramatic as those derived from A–T patients (Pan *et al*. 2002; table 1). In contrast to A–T and NBS patients, normal immunoglobulin levels are observed in most ATLD patients described to date (Stewart *et al*. 1999; Delia *et al*. 2004; Fernet *et al*. 2005). Significantly reduced numbers of Sμ–Sα and Sμ–Sγ clones have, however, also been shown in ATLD patients (Lahdesmaki *et al*. 2004). There is a trend (not significant) in the use of longer microhomologies at the Sμ–Sα junctions but not in the Sμ–Sγ junctions. The dependence of microhomology at the Sμ–Sα or Sμ–Sγ junctions is thus A–T>NBS>ATLD>control.

The Sμ–Sα junctions obtained from NBS and ATLD patients are, however, clearly different from those in A–T patients, in at least two respects: first, the blunt end direct joining mechanism (requiring no microhomology) is severely impaired in A–T patients but not in NBS or ATLD patients. Second, the precise joining of complementary DNA ends with 1–3 bp sequence homology, probably executed through a simple religation mechanism (Haber 1999), is normal in A–T patients, but defective in NBS and, to a lesser degree, ATLD patients. Mutations or insertions often occur at junctions with 1–3 bp microhomology in NBS and ATLD patients (Lahdesmaki *et al*. 2004). Thus, as in cells from A–T patients, the balance between the predominantly used NHEJ and the A-EJ pathways is altered. ATM and the Mre11 complex may, however, act on the predominant end-joining pathway in a different manner or at a different step (Pan *et al*. 2002; Lahdesmaki *et al*. 2004). NBS1 and Mre11 may also have roles in CSR, which are independent of each other, as the patterns of mutations and insertions around the Sμ–Sα junctions are clearly different. In NBS patients, all base substitutions were located at G/C nucleotides, and in ATLD patients, the substitutions that occurred most often in controls, C to T transitions, were never observed (Lahdesmaki *et al*. 2004). These peculiar patterns of base substitutions are, however, specific for the CSR reactions, as they are quite different from the SHM patterns we observed in the V_H_ regions from these patients (Du *et al*. 2008).

In mice, disruption of any subunit of the Mre11 complex results in embryonic lethality (Xiao & Weaver 1997; Luo *et al*. 1999; Zhu *et al*. 2001). A humanized mouse model for NBS has been generated by the introduction of the human 5 bp deletion hypomorphic allele into NBS-deficient mice (Difilippantonio *et al*. 2002). No CSR defect (to IgG1 and IgG3) has, however, been observed in this mouse model. Using a conditional knockout strategy, two studies have shown that CSR to various IgG subclasses is reduced in NBS1-deficient B cells (to approx. 50% of wild-type levels; Kracker *et al*. 2005; Reina-San-Martin *et al*. 2005). The CSR defect is not dependent on GL transcription and appears to be due to inefficient recombination at the DNA level (Reina-San-Martin *et al*. 2005). In both studies, a normal length of microhomology and a normal rate of mutations were found at the Sμ–Sγ junctions. It is noteworthy that in one of the studies, residual levels of wild-type NBS (25%) remained in the cells, and thus the effect of NBS1 on CSR may well have been underestimated (Kracker *et al*. 2005) as some CSR junctions might be derived from NBS-proficient cells. The level of IgA switching and the quality of Sμ–Sα junctions have not been analysed to date in these mice.

#### (vi) ATR

As discussed earlier, the CSR defect in 53BP1-deficient cells is even more severe than that in ATM-deficient cells. Another possibility is that additional upstream PIKKs, which activate 53BP1, might be involved in CSR. The A–T and Rad3-related protein (ATR) could be one such candidate, as it is closely related to ATM and targets an overlapping set of substrates, including 53BP1 and H2AX (Abraham 2001; Cimprich & Cortez 2008). Loss of ATR in mice results in embryonic lethality (Brown & Baltimore 2000) whereas hypomorphic mutations in the *ATR* gene have been identified in a few patients with the Seckel syndrome (O'Driscoll *et al*. 2003). These patients are characterized by intrauterine growth retardation, dwarfism, microcephaly, ‘bird-like’ facial features and mental retardation but no obvious immunodeficiency. Studies on B cells from these patients have shown that the proportion that has switched to IgA and IgG in the peripheral blood is normal (Pan-Hammarstrom *et al*. 2006). An analysis of the Sμ–Sα junctions showed a normal ‘blunt end-joining’ and a normal ‘simple religation’ process (joining 1–3 bp complementary ends), but impaired end joining with partially complementary (1–3 bp) DNA ends. There is also a significant increase in the length of microhomology at the Sμ–Sα junctions (3.0 bp versus 1.8 bp in controls), but only up to 9 bp. Thus, ATR is likely to have a moderate role in the predominant end-joining process during CSR. Whether this is due to its interaction with 53BP1 or γH2AX is, however, unknown. An additional speculation is that ATR may also be required in the A-EJ pathway where a longer microhomology (10 bp or more) is required (figure 2).

### (b) The classical non-homologous end joining machinery

#### (i) Ku70/Ku80

Ku is a heterodimer of Ku70 and Ku80. They are probably among the first proteins that bind to the DNA ends at a DSB and the Ku–DNA complex recruits and activates DNA-PKcs (Lieber *et al*. 2003; Lieber 2008). Ku and DNA-PKcs are proposed to act in the synapsis process (Lieber *et al*. 2003; Lieber 2008). Ku70 or Ku80 knockout mice are growth retarded, radiosensitive and are severely immunodeficient (Nussenzweig *et al*. 1996; Gu *et al*. 1997). B-cell development is arrested at an early stage due to a profound deficiency in V(D)J recombination (Nussenzweig *et al*. 1996; Gu *et al*. 1997). To assess the role of Ku in CSR, pre-rearranged *V*(*D*)*J heavy chain* and *VJ light chain* genes were introduced into a Ku70- or Ku80-deficient background. In both cases, CSR to IgG and/or IgE is severely impaired, suggesting that the Ku heterodimer is required for CSR (Casellas *et al*. 1998; Manis *et al*. 1998). However, the severe proliferation defect of Ku-deficient B cells has made it difficult to determine whether Ku, and thus NHEJ, has a direct role in end joining during CSR. No CSR junctions have been analysed in Ku70- or Ku80-deficient mice. However, the majority of the recovered coding or RS joints from Ku70-deficient cells exhibited 1–5 bp short homologies (Gu *et al*. 1997).

#### (ii) DNA-PKcs

The first piece of evidence that DNA-PKcs might be involved in CSR came from a study using pre-B cell lines derived from severe combined immunodeficiency (*Scid*) mice, where the ability of these lines' switch to IgE was impaired (Rolink *et al*. 1996). These mice carry a spontaneous point mutation in the *DNA-PKcs* gene, which results in a truncated protein lacking DNA-PKcs kinase activity (Blunt *et al*. 1996; Danska *et al*. 1996; Beamish *et al*. 2000). In a second study, Ig heavy and light chain knock-in DNA-PKcs-null mice were analysed and, despite a normal proliferation, GL transcription and AID induction, CSR to all the Ig classes was severely impaired, with the exception of IgG1, providing convincing evidence that DNA-PKcs is required for CSR (Manis *et al*. 2002). The importance of DNA-PKcs in CSR has, however, been challenged by studies where Ig heavy and light chain knock-in *Scid* (Bosma *et al*. 2002; Cook *et al*. 2003) or DNA-PKcs C-terminal deleted mice (Kiefer *et al*. 2007) were analysed. CSR efficiency was either close to normal (Kiefer *et al*. 2007) or reduced approximately two- to threefold (Bosma *et al*. 2002; Cook *et al*. 2003). The difference between the DNA-PKcs-null and *Scid* mice may suggest that the kinase activity of DNA-PKcs is not essential, or at least replaceable, during CSR, and DNA-PKcs may have a non-catalytic role in CSR, for instance by mediating synapse complex formation (DeFazio *et al*. 2002).

Based on the *Scid* studies, one can imagine a DNA-PKcs kinase-independent end-joining process in CSR. It would be of interest to know whether this is still part of NHEJ or it is the A-EJ used in the absence of ATM. In DNA-PKcs-null cells, the Sμ–Sγ1 junctions appear to be indistinguishable from controls (Manis *et al*. 2002). In one of the studies on *Scid* mice, CSR junctions have been analysed and a small increase in microhomology usage has been observed (3.4 bp versus 2.3 bp; Cook *et al*. 2003). However, this was based on a mixture of junctions (Sμ–Sγ, Sγ–Sϵ and Sμ–Sϵ). As Sμ–Sϵ junctions, similar to Sμ–Sα, tend to show longer microhomologies when NHEJ is impaired (Pan-Hammarstrom *et al*. 2005; Yan *et al*. 2007), this analysis is not conclusive. A separate analysis of different types of CSR junctions would be preferable. The pattern of mutations around these CSR junctions is, however, altered, with a significantly reduced number of C mutations (resembling those from ATLD patients), suggesting that the kinase activity of DNA-PKcs may have a role in the later steps of the end-joining process during CSR. This notion is also supported by a recent study showing that DNA-PKcs is required for end joining of a subset of AID-induced CSR breaks (Franco *et al*. 2008).

#### (iii) DNA ligase IV and XRCC4

The XRCC4–DNA ligase IV complex is responsible for catalysing the ligation step of NHEJ (Lieber *et al*. 2003; Lieber 2008). No human disease has yet been shown to be associated with a mutation in the *XRCC4* gene, whereas a few patients with defective DNA ligase IV activity have been described. These patients are characterized by microcephaly, growth retardation, radiosensitivity and mild to severe immunodeficiency (O'Driscoll *et al*. 2001; Buck *et al*. 2006; Enders *et al*. 2006; van der Burg *et al*. 2006). CSR junctions have been characterized in some of these patients and a marked shift to the usage of microhomology at the Sμ–Sα junctions has been observed (9.8 bp versus 1.8 bp in controls; Pan-Hammarstrom *et al*. 2005). This shift is most dramatic among all the patient groups that we have tested to date (DNA ligase IV>ATM>NBS>ATR>ATLD). Almost all junctions (97%) displayed at least 1 bp microhomology, the remaining being 1 bp insertions, thus the ‘blunt end joining’ or ‘direct end joining’ is completely abolished in the DNA ligase IV-deficient cells. Forty-seven per cent of the junctions exhibited a microhomology of 10 bp or more with the longest being 29 bp, which is by far the longest that has been described in human cells. A reduction of mutations or insertions at, or around, the Sμ–Sα junctions was also observed.

Despite the dramatic increase of microhomology at the Sμ–Sα junctions, the Sμ–Sγ junctions only show an increased frequency of insertions, but no significant increase in the length of junctional homology or rate of mutations (Pan-Hammarstrom *et al*. 2005). This difference cannot solely be due to differences in the homology between the Sμ and the Sα or Sγ regions, as a small, albeit significant, increased length of microhomology has been observed in the Sμ–Sγ junctions from ATM-, ATR- and NBS-deficient cells. Furthermore, no significant increase in the rate of insertions has been observed at the Sμ–Sγ junctions from these patients. It is possible that yet another alternative pathway is used for joining the Sμ–Sγ regions in the DNA ligase IV-deficient patients, where 1 bp insertions are frequently introduced. It is also possible that the residual level of DNA ligase IV activity in the patient cells results in different modes of CSR junction resolution.

Disruption of *LIG4* or *XRCC4* in mice results in embryonic lethality. Models that are based on XRCC4-deficient-mouse B cells have therefore been generated by two independent groups using different strategies (Soulas-Sprauel *et al*. 2007; Yan *et al*. 2007). CSR to IgG or IgE is reduced to approximately 20–50% of control levels in XRCC4-deficient cells (Soulas-Sprauel *et al*. 2007; Yan *et al*. 2007). In one of the studies, Yan *et al*. (2007) analysed Sμ–Sγ and Sμ–Sϵ junctions and demonstrated that there is a shift towards microhomology-based, alternative joining in the absence of XRCC4. They also found that ‘blunt’ or ‘direct’ end joining is totally abolished in XRCC4-deficient cells. In their preliminary studies, they have also observed a similar lack of direct joining and a bias towards a microhomology-based joining in DNA ligase IV-deficient mouse B cells. These results, taken together with the data obtained from DNA ligase IV-deficient patients, clearly suggest that the classical NHEJ machinery is required for CSR and, in its absence, an A-EJ mechanism is operating.

### (c) Additional factors

Increased microhomology at the CSR junctions has also been observed in mice deficient in PMS2, MLH1, MSH4 or MSH5 and in IgAD and common variable immunodeficiency (CVID) patients carrying mutations in the *MSH5* gene (Ehrenstein *et al*. 2001; Schrader *et al*. 2002; Sekine *et al*. 2007; Pan-Hammarstrom & Hammarstrom 2008). How the different mismatch repair pathways are connected to the NHEJ machinery is currently unclear. In addition to the patient who suffered from the RIDDLE syndrome, a marked shift towards microhomology at the Sμ–Sα junctions has also been observed in several patients suffering from another rare form of immunodeficiency (hyper IgM syndrome) with an unknown genetic basis (Peron *et al*. 2007). The predominant NHEJ pathway in CSR is thus likely to be regulated by additional DNA-damage response/repair factors that remain to be characterized.

## 3. The alternative end-joining pathway during class switch recombination

The A-EJ mechanism used in CSR and V(D)J recombination (Corneo *et al*. 2007) also contributes to the chromosomal translocations that give rise to lymphoid malignancies (Nussenzweig & Nussenzweig 2007; Yan *et al*. 2007). Compared with the predominant NHEJ pathway, however, we know rather little about the mechanism underlying A-EJ. Deletions, insertions and microhomologies are normally associated with the A-EJ pathway; however, we do not know the kinetics of the repair (fast or slow), during which cell cycle it is operative (G1 or others), how long a microhomology that is required (more than 1 bp, or 4 bp or even longer), whether there is only one or multiple pathways or the identity of the factors actually involved in the A-EJ mechanism. The identification of the factors in the A-EJ pathway may be complicated by the possibility that a short microhomology could be the result of either the NHEJ or A-EJ pathways, whereas lack of microhomology may not necessarily exclude the involvement of A-EJ. Furthermore, some factors regulating the classical NHEJ pathway may also be involved in the A-EJ process. A number of potential factors of the latter will be briefly discussed below.

### (a) The Mre11–Rad50–NBS1 complex

The 3′–5′ exonuclease activity of Mre11 has previously been suggested to be involved in microhomology-based end joining, where it degrades the mismatched DNA ends until sequence identity is revealed (Paull & Gellert 2000). Based on the CSR junctions from ATLD and NBS patients, we have previously hypothesized that the Mre11 complex may be required for repairing DNA ends with a short (1–3 bp) homology, which is likely to be part of the NHEJ reaction. The Mre11 complex is, however, unlikely to be involved in A-EJ, if this pathway requires microhomology of 4 bp or longer, as the proportion of junctions with these longer microhomologies is slightly increased, rather than decreased, in both patient groups (Lahdesmaki *et al*. 2004).

### (b) Poly (ADP-ribose) polymerase 1, XRCC1 and DNA ligase III

Poly (ADP-ribose) polymerase 1 (PARP-1) is an abundant nuclear protein that binds to both single-strand breaks (SSBs) and DSBs. It is involved in many cellular responses including base excision repair (BER) and, possibly, HR (Helleday *et al*. 2005). Together with XRCC1 and DNA ligase III, it has also been proposed as a candidate factor for the alternative, or back-up, pathway of NHEJ (Audebert *et al*. 2004; Wang *et al*. 2005, 2006). PARP-1 binds to DNA ends and, under normal conditions, it is in direct competition with Ku and possibly also DNA-PKcs (Wang *et al*. 2006). Inhibitors of PARP-1 have been found to increase IgA switching in a B cell line and enhance IgG1 switching in activated splenic B cells (Shockett & Stavnezer 1993). This may imply that PARP-1 would normally inhibit or compete with other factors that participate in the predominant NHEJ pathway during CSR, which fits well with the hypothesis that it may be a component of the A-EJ pathway. Inhibitors of PARP-1 can in fact lead to the activation of ATM in other DNA repair process such as HR (Bryant & Helleday 2006). It would therefore be of considerable interest to test the effect of PARP-1 in CSR on an NHEJ-deficient background. Both ligases I and III seem to be required for microhomology-mediated end joining (Liang *et al*. 2008) but which of the two ligases is involved in A-EJ during CSR remains to be clarified.

### (c) Werner protein

Werner protein (WRN) belongs to the RecQ helicase family and is mutated in the premature ageing disease, Werner's syndrome (Yu *et al*. 1996). WRN has been implicated in HR but has also been linked to NHEJ, as its exonuclease activity is stimulated by Ku70/Ku80 (Li & Comai 2000). WRN, together with ligase III, has recently been implicated in A-EJ activity in chronic myeloid leukaemia cells, where knockdown of these factors leads to a significant reduction of microhomology at the repair sites (Sallmyr *et al*. 2008). However, WRN physically interacts with one of the key NHEJ factors, the XRCC4–DNA ligase IV complex, which stimulates WRN's exonuclease activity (Kusumoto *et al*. 2008). Thus, it is unclear which pathway WRN is actually involved in during CSR (if it has a role at all).

## 4. Concluding remarks

Over the past decade, we have begun to understand the complex molecular mechanisms underlying DNA editing, repair and recombination during CSR. Two major repair pathways have been proposed, the predominant NHEJ and the A-EJ involving microhomologies (figure 2). A more complicated picture will probably emerge when the NHEJ pathway is further dissected and the A-EJ pathway is delineated. The ultimate goal will be to have a three-dimensional dynamic model for CSR, where we would know the molecular basis of the process and the interaction between the DNA repair factors at the switch regions.

## Figures and Tables

**Table 1:** CSR phenotype in cells deficient for various DNA repair factors that might be involved in the NHEJ pathway during CSR.

protein	model	CSR efficiency	proliferation defect	GL transcription	type of junctions	significant shift towards use of microhomology	frequency of junctional mutations^a^	references
ATM	human	reduced (IgA)	n.a.^b^	n.a.	Sμ–Sα	yes (7.2 versus 1.8 bp)	reduced	Pan *et al*. (2002)
					Sμ–Sγ	yes (2.5 versus 1.2 bp)	reduced	
	mouse	reduced (IgA, IgG1, IgG2a, IgG3, IgE)	no	normal	Sμ–Sγ1	yes (2.6 versus 1.2 bp)	n.a.	Lumsden *et al*. (2004)
		7–50% of controls						
	mouse	reduced (IgG1, IgG2b, IgG3)	no	normal	Sμ–Sγ1	no (1.9 versus 2.0 bp)	reduced	Reina-San-Martin *et al*. (2004)
		30% of controls						
H2AX	mouse	reduced (IgG1)	no	normal	n.a.	n.a.	n.a.	Petersen *et al*. (2001)
		24–50% of controls						
	mouse	reduced (IgG3)	no	n.a.	n.a.	n.a.	n.a.	Celeste *et al*. (2002)
		30% of controls						
	mouse	reduced (IgG1)	no	normal	Sμ–Sγ1	no (1.8 versus 1.7 bp)	reduced (n.s.)^c^	Reina-San-Martin *et al*. (2003)
MDC1	mouse	reduced (IgG1)	no	n.a.	n.a.	n.a.	n.a.	Lou *et al*. (2006)
		50–75% of controls						
53BP1	mouse	reduced (IgG1)	no	normal	n.a.	n.a.	n.a.	Ward *et al*. (2004)
		7% of controls						
	mouse	reduced (all Ig classes)	no	normal	Sμ–Sγ1	no (0.9 versus 1.1 bp)	reduced	Manis *et al*. (2004)
		2–10% of controls						
	mouse	n.a.	n.a.	n.a.	Sμ–Sγ1	no (2.5 versus 2.0 bp)	normal	Reina-San-Martin *et al*. (2007)
NBS	human	reduced (IgA and IgG)	n.a.	n.a.	Sμ–Sα	yes (3.6 versus 1.8 bp)	normal	Pan *et al*. (2002) and Lahdesmaki *et al*. (2004)
					Sμ–Sγ	yes (2.3 versus 1.2 bp)	normal	
	mouse	reduced (IgG1 and IgG3)	no	normal	Sμ–Sγ1	no (2.9 versus 2.3 bp)	normal	Kracker *et al*. (2005)
		approx. 50% of controls						
	mouse	reduced (IgG1)	yes	normal	Sμ–Sγ1	no (1.4 versus 0.8 bp)	normal	Reina-San-Martin *et al*. (2005)
		approx. 50% of controls						
Mre11	human	reduced (IgA and IgG)	n.a.	n.a.	Sμ–Sα	no (2.6 versus 1.8 bp)	reduced C to T	Lahdesmaki *et al*. (2004)
					Sμ–Sγ	no (1.8 versus 1.2 bp)	n.a.	
ATR	human	normal (IgA and IgG)	n.a.	n.a.	Sμ–Sα	yes (3.0 versus 1.8 bp)	reduced	Pan-Hammarstrom *et al*. (2006)
					Sμ–Sγ	yes (1.8 versus 1.2 bp)	reduced (n.s.)	
Ku70	mouse	impaired (IgG1, IgG2b, IgG3 and IgE) not detectable	yes	normal	n.a.	n.a.	n.a.	Manis *et al*. (1998)
Ku80	mouse	impaired (IgG1 and IgG3)	yes	normal	n.a.	n.a.	n.a.	Casellas *et al*. (1998)
		not detectable						
DNA-PKcs	mouse	reduced (IgE)	no	normal	n.a.	n.a.	n.a.	Rolink *et al*. (1996)
		100- to 250-fold less						
	mouse	impaired (all the isotypes except IgG1) not detectable	no	normal	Sμ–Sγ1	no	n.a.	Manis *et al*. (2002)
	mouse	reduced (IgA, IgG1, IgG3 and IgG2b)	no	n.a.	n.a.	n.a.	n.a.	Bosma *et al*. (2002)
		30–50% of controls						
	mouse	reduced (IgG1, IgG2a, IgG2b, IgG3, IgE)	yes	n.a.	Sμ–SγSμ–Sϵ	yes (3.4 versus 2.3 bp)	reduced(reduced C)	Cook *et al*. (2003)
		40–70% of controls						
	mouse	near normal (IgG2b, IgG3, and IgA)	n.a.	n.a.	n.a.	n.a.	n.a.	Kiefer *et al*. (2007)
DNA ligase IV	human	reduced (IgA and IgG)	n.a.	n.a.	Sμ–Sα	yes (9.8 versus 1.8 bp)	reduced	Pan-Hammarstrom *et al*. (2005)
					Sμ–Sγ	no (1.3 versus 1.2 bp)	normal	
	mouse	reduced (IgG1 and IgE)	n.a.	n.a.	?	yes	n.a.	Yan *et al*. (2007)
		50% of controls						
XRCC4	mouse	reduced (IgG1 and IgG3)	no	yes	Sμ–Sγ1	no	n.a.	Soulas-Sprauel *et al*. (2007)
		40–75% of controls						
	mouse	reduced (IgG1 and IgG3)	no	n.a.	Sμ–Sγ	yes	reduced	Yan *et al*. (2007)
		20–50% of controls			Sγ–Sϵ			
					Sμ–Sϵ			

a Mutations around junctions, ±15 bp for human study and ±50 bp for mouse study.

b n.a., not analysed.

c n.s., not significant.

## References

[bib1] Abraham R.T. (2001). Cell cycle checkpoint signaling through the ATM and ATR kinases. Genes Dev.

[bib2] Audebert M., Salles B., Calsou P. (2004). Involvement of poly(ADP-ribose) polymerase-1 and XRCC1/DNA ligase III in an alternative route for DNA double-strand breaks rejoining. J. Biol. Chem.

[bib3] Barlow C. (1996). Atm-deficient mice: a paradigm of ataxia telangiectasia. Cell.

[bib4] Beamish H.J., Jessberger R., Riballo E., Priestley A., Blunt T., Kysela B., Jeggo P.A. (2000). The C-terminal conserved domain of DNA-PKcs, missing in the SCID mouse, is required for kinase activity. Nucleic Acids Res.

[bib5] Berkovich E., Monnat R.J., Kastan M.B. (2007). Roles of ATM and NBS1 in chromatin structure modulation and DNA double-strand break repair. Nat. Cell Biol.

[bib6] Blunt T., Gell D., Fox M., Taccioli G.E., Lehmann A.R., Jackson S.P., Jeggo P.A. (1996). Identification of a nonsense mutation in the carboxyl-terminal region of DNA-dependent protein kinase catalytic subunit in the scid mouse. Proc. Natl Acad. Sci. USA.

[bib7] Bosma G.C., Kim J., Urich T., Fath D.M., Cotticelli M.G., Ruetsch N.R., Radic M.Z., Bosma M.J. (2002). DNA-dependent protein kinase activity is not required for immunoglobulin class switching. J. Exp. Med.

[bib8] Brown E.J., Baltimore D. (2000). ATR disruption leads to chromosomal fragmentation and early embryonic lethality. Genes Dev.

[bib9] Bryant H.E., Helleday T. (2006). Inhibition of poly (ADP-ribose) polymerase activates ATM which is required for subsequent homologous recombination repair. Nucleic Acids Res.

[bib10] Buck D. (2006). Severe combined immunodeficiency and microcephaly in siblings with hypomorphic mutations in DNA ligase IV. Eur. J. Immunol.

[bib11] Burma S., Chen B.P., Murphy M., Kurimasa A., Chen D.J. (2001). ATM phosphorylates histone H2AX in response to DNA double-strand breaks. J. Biol. Chem.

[bib12] Casellas R. (1998). Ku80 is required for immunoglobulin isotype switching. EMBO J.

[bib13] Celeste A. (2002). Genomic instability in mice lacking histone H2AX. Science.

[bib14] Chaudhuri J., Alt F.W. (2004). Class-switch recombination: interplay of transcription, DNA deamination and DNA repair. Nat. Rev. Immunol.

[bib15] Chun H.H., Gatti R.A. (2004). Ataxia–telangiectasia, an evolving phenotype. DNA Repair.

[bib16] Cimprich K.A., Cortez D. (2008). ATR: an essential regulator of genome integrity. Nat. Rev. Mol. Cell Biol.

[bib17] Cook A.J., Oganesian L., Harumal P., Basten A., Brink R., Jolly C.J. (2003). Reduced switching in SCID B cells is associated with altered somatic mutation of recombined S regions. J. Immunol.

[bib18] Corneo B. (2007). Rag mutations reveal robust alternative end joining. Nature.

[bib19] D'Amours D., Jackson S.P. (2002). The Mre11 complex: at the crossroads of DNA repair and checkpoint signalling. Nat. Rev. Mol. Cell Biol.

[bib20] Danska J.S., Holland D.P., Mariathasan S., Williams K.M., Guidos C.J. (1996). Biochemical and genetic defects in the DNA-dependent protein kinase in murine scid lymphocytes. Mol. Cell Biol.

[bib21] DeFazio L.G., Stansel R.M., Griffith J.D., Chu G. (2002). Synapsis of DNA ends by DNA-dependent protein kinase. EMBO J.

[bib22] Delia D., Piane M., Buscemi G., Savio C., Palmeri S., Lulli P., Carlessi L., Fontanella E., Chessa L. (2004). MRE11 mutations and impaired ATM-dependent responses in an Italian family with ataxia–telangiectasia-like disorder. Hum. Mol. Genet.

[bib23] Difilippantonio M.J., Petersen S., Chen H.T., Johnson R., Jasin M., Kanaar R., Ried T., Nussenzweig A. (2002). Evidence for replicative repair of DNA double-strand breaks leading to oncogenic translocation and gene amplification. J. Exp. Med.

[bib24] Digweed M., Sperling K. (2004). Nijmegen breakage syndrome: clinical manifestation of defective response to DNA double-strand breaks. DNA Repair.

[bib25] DiTullio R.A., Mochan T.A., Venere M., Bartkova J., Sehested M., Bartek J., Halazonetis T.D. (2002). 53BP1 functions in an ATM-dependent checkpoint pathway that is constitutively activated in human cancer. Nat. Cell Biol.

[bib26] Du L. (2008). A regulatory role for NBS1 in strand-specific mutagenesis during somatic hypermutation. PLoS ONE.

[bib27] Ehrenstein M.R., Rada C., Jones A.M., Milstein C., Neuberger M.S. (2001). Switch junction sequences in PMS2-deficient mice reveal a microhomology-mediated mechanism of Ig class switch recombination. Proc. Natl Acad. Sci. USA.

[bib28] Elson A., Wang Y., Daugherty C.J., Morton C.C., Zhou F., Campos-Torres J., Leder P. (1996). Pleiotropic defects in ataxia–telangiectasia protein-deficient mice. Proc. Natl Acad. Sci. USA.

[bib29] Enders A. (2006). A severe form of human combined immunodeficiency due to mutations in DNA ligase IV. J. Immunol.

[bib30] Fernandez-Capetillo O. (2002). DNA damage-induced G2-M checkpoint activation by histone H2AX and 53BP1. Nat. Cell Biol.

[bib31] Fernet M., Gribaa M., Salih M.A., Seidahmed M.Z., Hall J., Koenig M. (2005). Identification and functional consequences of a novel MRE11 mutation affecting 10 Saudi Arabian patients with the ataxia telangiectasia-like disorder. Hum. Mol. Genet.

[bib32] Franco S. (2006). H2AX prevents DNA breaks from progressing to chromosome breaks and translocations. Mol. Cell.

[bib33] Franco S., Murphy M.M., Li G., Borjeson T., Boboila C., Alt F.W. (2008). DNA-PKcs and Artemis function in the end-joining phase of immunoglobulin heavy chain class switch recombination. J. Exp. Med.

[bib34] Goldberg M., Stucki M., Falck J., D'Amours D., Rahman D., Pappin D., Bartek J., Jackson S.P. (2003). MDC1 is required for the intra-S-phase DNA damage checkpoint. Nature.

[bib35] Gregorek H., Chrzanowska K.H., Michalkiewicz J., Syczewska M., Madalinski K. (2002). Heterogeneity of humoral immune abnormalities in children with Nijmegen breakage syndrome: an 8-year follow-up study in a single centre. Clin. Exp. Immunol.

[bib36] Gu Y. (1997). Growth retardation and leaky SCID phenotype of Ku70-deficient mice. Immunity.

[bib37] Haber J.E. (1999). Sir-Ku-itous routes to make ends meet. Cell.

[bib38] Helleday T., Bryant H.E., Schultz N. (2005). Poly(ADP-ribose) polymerase (PARP-1) in homologous recombination and as a target for cancer therapy. Cell Cycle.

[bib39] Hickson I. (2004). Identification and characterization of a novel and specific inhibitor of the ataxia–telangiectasia mutated kinase ATM. Cancer Res.

[bib40] Islam K.B., Baskin B., Nilsson L., Hammarström L., Sideras P., Smith C.I. (1994). Molecular analysis of IgA deficiency. Evidence for impaired switching to IgA. J. Immunol.

[bib41] Iwabuchi K., Bartel P.L., Li B., Marraccino R., Fields S. (1994). Two cellular proteins that bind to wild-type but not mutant p53. Proc. Natl Acad. Sci. USA.

[bib42] Kiefer K., Oshinsky J., Kim J., Nakajima P.B., Bosma G.C., Bosma M.J. (2007). The catalytic subunit of DNA-protein kinase (DNA-PKcs) is not required for Ig class-switch recombination. Proc. Natl Acad. Sci. USA.

[bib43] Kracker S. (2005). Nibrin functions in Ig class-switch recombination. Proc. Natl Acad. Sci. USA.

[bib44] Kusumoto R., Dawut L., Marchetti C., Wan Lee J., Vindigni A., Ramsden D., Bohr V.A. (2008). Werner protein cooperates with the XRCC4-DNA ligase IV complex in end-processing. Biochemistry.

[bib45] Lahdesmaki A., Taylor A.M., Chrzanowska K.H., Pan-Hammarstrom Q. (2004). Delineation of the role of the Mre11 complex in class switch recombination. J. Biol. Chem.

[bib47] Lavin M.F. (2007). ATM and the Mre11 complex combine to recognize and signal DNA double-strand breaks. Oncogene.

[bib48] Lavin M.F., Kozlov S. (2007). ATM activation and DNA damage response. Cell Cycle.

[bib46] Lavin M., Shiloh Y., Ochs H.D., Smith C.I.E., Puck J.M. (1999). Ataxia–telangiectasia. Primary immunodeficiency diseases.

[bib49] Li B., Comai L. (2000). Functional interaction between Ku and the Werner syndrome protein in DNA end processing. J. Biol. Chem.

[bib50] Liang L., Deng L., Nguyen S.C., Zhao X., Maulion C.D., Shao C., Tischfield J.A. (2008). Human DNA ligases I and III, but not ligase IV, are required for microhomology-mediated end joining of DNA double-strand breaks. Nucleic Acids Res.

[bib51] Lieber M.R. (1999). The biochemistry and biological significance of nonhomologous DNA end joining: an essential repair process in multicellular eukaryotes. Genes Cells.

[bib52] Lieber M.R. (2008). The mechanism of human nonhomologous DNA end joining. J. Biol. Chem.

[bib53] Lieber M.R., Ma Y., Pannicke U., Schwarz K. (2003). Mechanism and regulation of human non-homologous DNA end-joining. Nat. Rev. Mol. Cell Biol.

[bib55] Lou Z., Minter-Dykhouse K., Wu X., Chen J. (2003). MDC1 is coupled to activated CHK2 in mammalian DNA damage response pathways. Nature.

[bib54] Lou Z. (2006). MDC1 maintains genomic stability by participating in the amplification of ATM-dependent DNA damage signals. Mol. Cell.

[bib56] Lumsden J.M. (2004). Immunoglobulin class switch recombination is impaired in Atm-deficient mice. J. Exp. Med.

[bib57] Luo G., Yao M.S., Bender C.F., Mills M., Bladl A.R., Bradley A., Petrini J.H. (1999). Disruption of mRad50 causes embryonic stem cell lethality, abnormal embryonic development, and sensitivity to ionizing radiation. Proc. Natl Acad. Sci. USA.

[bib59] Manis J.P., Gu Y., Lansford R., Sonoda E., Ferrini R., Davidson L., Rajewsky K., Alt F.W. (1998). Ku70 is required for late B cell development and immunoglobulin heavy chain class switching. J. Exp. Med.

[bib58] Manis J.P., Dudley D., Kaylor L., Alt F.W. (2002). IgH class switch recombination to IgG1 in DNA-PKcs-deficient B cells. Immunity.

[bib60] Manis J.P., Morales J.C., Xia Z., Kutok J.L., Alt F.W., Carpenter P.B. (2004). 53BP1 links DNA damage-response pathways to immunoglobulin heavy chain class-switch recombination. Nat. Immunol.

[bib61] Matsuoka S. (2007). ATM and ATR substrate analysis reveals extensive protein networks responsive to DNA damage. Science.

[bib62] Mills F.C., Brooker J.S., Camerini-Otero R.D. (1990). Sequences of human immunoglobulin switch regions: implications for recombination and transcription. Nucleic Acids Res.

[bib63] Mochan T.A., Venere M., DiTullio R.A., Halazonetis T.D. (2004). 53BP1, an activator of ATM in response to DNA damage. DNA Repair.

[bib64] Muramatsu M., Kinoshita K., Fagarasan S., Yamada S., Shinkai Y., Honjo T. (2000). Class switch recombination and hypermutation require activation-induced cytidine deaminase (AID), a potential RNA editing enzyme. Cell.

[bib66] Nussenzweig A., Nussenzweig M.C. (2007). A backup DNA repair pathway moves to the forefront. Cell.

[bib65] Nussenzweig A., Chen C., da Costa Soares V., Sanchez M., Sokol K., Nussenzweig M.C., Li G.C. (1996). Requirement for Ku80 in growth and immunoglobulin V(D)J recombination. Nature.

[bib67] O'Driscoll M. (2001). DNA ligase IV mutations identified in patients exhibiting developmental delay and immunodeficiency. Mol. Cell.

[bib68] O'Driscoll M., Ruiz-Perez V.L., Woods C.G., Jeggo P.A., Goodship J.A. (2003). A splicing mutation affecting expression of ataxia–telangiectasia and Rad3-related protein (ATR) results in Seckel syndrome. Nat. Genet.

[bib69] Pan Q., Petit-Frere C., Dai S., Huang P., Morton H.C., Brandtzaeg P., Hammarstrom L. (2001). Regulation of switching and production of IgA in human B cells in donors with duplicated alpha1 genes. Eur. J. Immunol.

[bib70] Pan Q., Petit-Frere C., Lahdesmaki A., Gregorek H., Chrzanowska K.H., Hammarstrom L. (2002). Alternative end joining during switch recombination in patients with ataxia–telangiectasia. Eur. J. Immunol.

[bib71] Pan-Hammarstrom Q., Hammarstrom L. (2008). Antibody deficiency diseases. Eur. J. Immunol.

[bib72] Pan-Hammarstrom Q., Jones A.M., Lahdesmaki A., Zhou W., Gatti R.A., Hammarstrom L., Gennery A.R., Ehrenstein M.R. (2005). Impact of DNA ligase IV on nonhomologous end joining pathways during class switch recombination in human cells. J. Exp. Med.

[bib73] Pan-Hammarstrom Q. (2006). Disparate roles of ATR and ATM in immunoglobulin class switch recombination and somatic hypermutation. J. Exp. Med.

[bib74] Pan-Hammarstrom Q., Zhao Y., Hammarstrom L. (2007). Class switch recombination: a comparison between mouse and human. Adv. Immunol.

[bib75] Paull T.T., Gellert M. (2000). A mechanistic basis for Mre11-directed DNA joining at microhomologies. Proc. Natl Acad. Sci. USA.

[bib76] Peron S. (2007). A primary immunodeficiency characterized by defective immunoglobulin class switch recombination and impaired DNA repair. J. Exp. Med.

[bib77] Petersen S. (2001). AID is required to initiate Nbs1/gamma-H2AX focus formation and mutations at sites of class switching. Nature.

[bib80] Reina-San-Martin B., Difilippantonio S., Hanitsch L., Masilamani R.F., Nussenzweig A., Nussenzweig M.C. (2003). H2AX is required for recombination between immunoglobulin switch regions but not for intra-switch region recombination or somatic hypermutation. J. Exp. Med.

[bib78] Reina-San-Martin B., Chen H.T., Nussenzweig A., Nussenzweig M.C. (2004). ATM is required for efficient recombination between immunoglobulin switch regions. J. Exp. Med.

[bib81] Reina-San-Martin B., Nussenzweig M.C., Nussenzweig A., Difilippantonio S. (2005). Genomic instability, endoreduplication, and diminished Ig class-switch recombination in B cells lacking Nbs1. Proc. Natl Acad. Sci. USA.

[bib79] Reina-San-Martin B., Chen J., Nussenzweig A., Nussenzweig M.C. (2007). Enhanced intra-switch region recombination during immunoglobulin class switch recombination in 53BP1^−/−^ B cells. Eur. J. Immunol.

[bib82] Revy P. (2000). Activation-induced cytidine deaminase (AID) deficiency causes the autosomal recessive form of the Hyper-IgM syndrome (HIGM2). Cell.

[bib83] Riballo E. (2004). A pathway of double-strand break rejoining dependent upon ATM, Artemis, and proteins locating to gamma-H2AX foci. Mol. Cell.

[bib84] Rogakou E.P., Boon C., Redon C., Bonner W.M. (1999). Megabase chromatin domains involved in DNA double-strand breaks *in vivo*. J. Cell Biol.

[bib85] Rolink A., Melchers F., Andersson J. (1996). The SCID but not the RAG-2 gene product is required for Sm-Se heavy chain class switching. Immunity.

[bib86] Rooney S., Alt F.W., Sekiguchi J., Manis J.P. (2005). Artemis-independent functions of DNA-dependent protein kinase in Ig heavy chain class switch recombination and development. Proc. Natl Acad. Sci. USA.

[bib87] Sallmyr A., Tomkinson A.E., Rassool F.V. (2008). Up-regulation of WRN and DNA ligase IIIα in chronic myeloid leukemia: consequences for the repair of DNA double strand breaks. Blood.

[bib89] Schrader C.E., Vardo J., Stavnezer J. (2002). Role for mismatch repair proteins Msh2, Mlh1, and Pms2 in immunoglobulin class switching shown by sequence analysis of recombination junctions. J. Exp. Med.

[bib88] Schrader C.E., Guikema J.E., Linehan E.K., Selsing E., Stavnezer J. (2007). Activation-induced cytidine deaminase-dependent DNA breaks in class switch recombination occur during G1 phase of the cell cycle and depend upon mismatch repair. J. Immunol.

[bib90] Schultz L.B., Chehab N.H., Malikzay A., Halazonetis T.D. (2000). p53 binding protein 1 (53BP1) is an early participant in the cellular response to DNA double-strand breaks. J. Cell Biol.

[bib91] Sekine H. (2007). Role for Msh5 in the regulation of Ig class switch recombination. Proc. Natl Acad. Sci. USA.

[bib92] Shiloh Y. (2003). ATM and related protein kinases: safeguarding genome integrity. Nat. Rev. Cancer.

[bib93] Shockett P., Stavnezer J. (1993). Inhibitors of poly(ADP-ribose) polymerase increase antibody class switching. J. Immunol.

[bib94] Soulas-Sprauel P., Le Guyader G., Rivera-Munoz P., Abramowski V., Olivier-Martin C., Goujet-Zalc C., Charneau P., de Villartay J.P. (2007). Role for DNA repair factor XRCC4 in immunoglobulin class switch recombination. J. Exp. Med.

[bib95] Stavnezer J. (1996). Antibody class switching. Adv. Immunol.

[bib96] Stavnezer J., Guikema J.E., Schrader C.E. (2008). Mechanism and regulation of class switch recombination. Annu. Rev. Immunol.

[bib97] Stewart G.S. (1999). The DNA double-strand break repair gene hMRE11 is mutated in individuals with an ataxia–telangiectasia-like disorder. Cell.

[bib99] Stewart G.S., Wang B., Bignell C.R., Taylor A.M., Elledge S.J. (2003). MDC1 is a mediator of the mammalian DNA damage checkpoint. Nature.

[bib98] Stewart G.S. (2007). RIDDLE immunodeficiency syndrome is linked to defects in 53BP1-mediated DNA damage signaling. Proc. Natl Acad. Sci. USA.

[bib100] Stucki M., Jackson S.P. (2006). GammaH2AX and MDC1: anchoring the DNA-damage-response machinery to broken chromosomes. DNA Repair.

[bib101] van der Burg M. (2006). A new type of radiosensitive T-B-NK+ severe combined immunodeficiency caused by a LIG4 mutation. J. Clin. Invest.

[bib102] Varon R. (1998). Nibrin, a novel DNA double-strand break repair protein, is mutated in Nijmegen breakage syndrome. Cell.

[bib103] Wang B., Matsuoka S., Carpenter P.B., Elledge S.J. (2002). 53BP1, a mediator of the DNA damage checkpoint. Science.

[bib104] Wang H., Rosidi B., Perrault R., Wang M., Zhang L., Windhofer F., Iliakis G. (2005). DNA ligase III as a candidate component of backup pathways of nonhomologous end joining. Cancer Res.

[bib105] Wang M., Wu W., Wu W., Rosidi B., Zhang L., Wang H., Iliakis G. (2006). PARP-1 and Ku compete for repair of DNA double strand breaks by distinct NHEJ pathways. Nucleic Acids Res.

[bib106] Ward I.M., Minn K., van Deursen J., Chen J. (2003). p53 Binding protein 53BP1 is required for DNA damage responses and tumor suppression in mice. Mol. Cell Biol.

[bib107] Ward I.M. (2004). 53BP1 is required for class switch recombination. J. Cell Biol.

[bib108] Xiao Y., Weaver D.T. (1997). Conditional gene targeted deletion by Cre recombinase demonstrates the requirement for the double-strand break repair Mre11 protein in murine embryonic stem cells. Nucleic Acids Res.

[bib109] Xie A. (2007). Distinct roles of chromatin-associated proteins MDC1 and 53BP1 in mammalian double-strand break repair. Mol. Cell.

[bib110] Xu Y., Ashley T., Brainerd E.E., Bronson R.T., Meyn M.S., Baltimore D. (1996). Targeted disruption of ATM leads to growth retardation, chromosomal fragmentation during meiosis, immune defects, and thymic lymphoma. Genes Dev.

[bib111] Yan C.T. (2007). IgH class switching and translocations use a robust non-classical end-joining pathway. Nature.

[bib112] Yu C.E. (1996). Positional cloning of the Werner's syndrome gene. Science.

[bib113] Zhu J., Petersen S., Tessarollo L., Nussenzweig A. (2001). Targeted disruption of the Nijmegen breakage syndrome gene NBS1 leads to early embryonic lethality in mice. Curr. Biol.

